# A Systematic Assessment of Accuracy in Detecting Somatic Mosaic Variants by Deep Amplicon Sequencing: Application to *NF2* Gene

**DOI:** 10.1371/journal.pone.0129099

**Published:** 2015-06-12

**Authors:** Elisa Contini, Irene Paganini, Roberta Sestini, Luisa Candita, Gabriele Lorenzo Capone, Lorenzo Barbetti, Serena Falconi, Sabrina Frusconi, Irene Giotti, Costanza Giuliani, Francesca Torricelli, Matteo Benelli, Laura Papi

**Affiliations:** 1 Diagnostic Genetics Unit, Careggi University Hospital, Florence, Italy; 2 Department of Biomedical Experimental and Clinical Sciences, Medical Genetics, University of Florence, Florence, Italy; Mayo Clinic, UNITED STATES

## Abstract

The accurate detection of low-allelic variants is still challenging, particularly for the identification of somatic mosaicism, where matched control sample is not available. High throughput sequencing, by the simultaneous and independent analysis of thousands of different DNA fragments, might overcome many of the limits of traditional methods, greatly increasing the sensitivity. However, it is necessary to take into account the high number of false positives that may arise due to the lack of matched control samples. Here, we applied deep amplicon sequencing to the analysis of samples with known genotype and variant allele fraction (VAF) followed by a tailored statistical analysis. This method allowed to define a minimum value of VAF for detecting mosaic variants with high accuracy. Then, we exploited the estimated VAF to select candidate alterations in *NF2* gene in 34 samples with unknown genotype (30 blood and 4 tumor DNAs), demonstrating the suitability of our method. The strategy we propose optimizes the use of deep amplicon sequencing for the identification of low abundance variants. Moreover, our method can be applied to different high throughput sequencing approaches to estimate the background noise and define the accuracy of the experimental design.

## Introduction

Mosaicism has been defined as the simultaneous presence, in a single individual, of two or more cell populations with different genotypes arising from the same fertilized egg. It is involved in more than 30 monogenic disorders showing variable expressivity according to the amount of mutated cells [1]. The occurrence of mosaicism depends on the disorders and its real frequency could be underestimated because of ascertainment bias, i.e. a low level of mosaicism in leucocytes might remain undetected if the molecular analysis is limited to blood cell DNAs.

Mosaicism is a frequent occurrence in Neurofibromatosis type 2 (NF2, MIM 101000) [2–4], a tumor predisposition syndrome characterized by the development of bilateral vestibular schwannomas, often associated with other schwannomas of cranial and spinal nerves, as well as meningiomas and ependimomas [5–7].

NF2 is an autosomal dominant disorder caused by alterations in the *NF2* tumor suppressor gene (MIM 607379), located in 22q12 [8, 9]. Germline variants of *NF2* are detected in about 91% of familial cases and 45–50% of sporadic ones [2, 3]. Mosaicism has been confirmed in about 25–33% of de novo NF2 patients [2–4], with the alteration only detected in tumors and not in DNA from leukocytes, and it could be even more common in sporadic patients with mild phenotypes [4].

The identification of mosaicism is challenging, but it is a critical point for NF2 patients: the amount and the distribution of mutated cells affects the clinical phenotype, as well as the transmission risk to offspring. For these reasons, the development of analysis methods for the identification of low level of mutated alleles is really important. Until now only the identification of the same *NF2* variant in at least two tumors of the patient enables a diagnosis of mosaic NF2, even if the alteration is not detected in blood DNA.

Recently, alternative methods to increase the sensitivity in detecting low amount of mutated alleles have been developed. High Resolution Melting Analysis (HRMA) allows to identify alterations in the melting curve of amplicons even if the variants are unknown and represents a very sensitive approach to detect low percentages of mutated cells in blood DNA [10, 11]. However this approach does not permit to characterize the specific alteration. The variant must be identified by Sanger sequencing that has a detection limit of about 10% of mutated allele [11]. The alternative to clone the PCR product in plasmid vectors, amplifying the plasmids, and sequencing the individual clones is too laborious to be used in a routinely diagnostic practice. Digital PCR greatly increases the sensitivity in revealing low level of mutated alleles using an emulsion quantitative real time PCR with two fluorescent probes specific for wild type or mutated allele, respectively [12]; however, a priori knowledge of the variant is needed. Finally, COLD PCR [13] and ICE-COLD PCR [14] approaches were used to selectively amplify mutated alleles present in low percentage in genomic DNA. By using a lower denaturing temperature it is possible to preferentially denature and amplify the minority allele and detect it by direct sequencing of the corresponding amplicon even if its percentage is lower than 10% [15]. However, as for digital PCR, these techniques are strictly subordinate to the knowledge of position and type of the variant.

High throughput sequencing may overcome the problem. Over the last few years, next-generation sequencing (NGS) has become a popular strategy for genotyping, enabling more precise variant detection compared to traditional methods due to its high resolution and high throughput. The simultaneous and independent analysis of hundreds or thousands of DNA fragments, allows low abundance variants to become readily detectable; on the other hand, the identification of the exact type of alteration allows to use other techniques, such as digital, COLD and ICE-COLD PCRs, to verify the presence of such alterations.

Nevertheless, even with NGS, detecting mosaicism is still challenging, especially for low allelic-fraction variants.

Several factors affect the sensitivity and specificity of low level allelic-fraction variant calls along the genome, including the depth of sequence coverage, the local sequencing error rate, the allelic fraction of the variant and the evidence thresholds used to declare a variant [16]. The read depth necessary to accurately detect a variant is dependent on the prevalence of the mutant allele. Targeted panel sequencing, and in particular amplicon sequencing, has been widely used to increase single nucleotide variation (SNV) detection sensitivity by achieving a high median read depth, even on a bench top sequencer. At the moment this experimental design is the best suitable approach to meet the critical needs of high sensitivity and specificity in the detection of low level allelic-fraction [17, 18].

To date, specific bioinformatic methods for the detection of somatic mosaic variants have not been estabilished, although several methods have been developed to identify somatic variants in tumor tissues (which are, similarly to the mosaic ones, low level allelic fraction variants). In general, these methods belong to two groups: 1) independent analysis for tumor and normal datasets from an individual, 2) simultaneous analysis for matched tumor and normal datasets using joint probability-based statistical approaches.

The comparison of matched tumor and germline samples is crucial not only to distinguish somatic from germline variants but also to bring out low allelic-fraction from background noise caused by high sequencing error rate. So far, methods developed to identify tumor-associated variants have not been tested for the identification of somatic mosaicism, where matched control sample is absent. For this reason, the use of tumor-associated somatic variants detection methods in single sample mode leads to the identification of a large number of false positive events [16].

This manuscript describes the strategy used to identify low allelic-fraction variants in *NF2* gene by deep sequencing approach followed by tailored bioinformatic analysis.

## Materials and Methods

### Patients and Calibration samples

Constitutional DNA samples were obtained from peripheral blood leukocytes.

DNA was extracted from blood leukocytes and from fresh frozen tumor tissue using standard procedures with phenol/chloroform extraction and ammonium acetate/ethanol precipitation.

To evaluate the diagnostic sensitivity of Illumina Miseq platform and its ability to identify mosaicism in DNA samples, we performed serial dilutions of a wild-type DNA with genomic DNA deriving from five non-mosaic NF2 patients carrying five different *NF2* variants.

Each patient DNA sample was mixed with wild type DNA to obtain 3 calibration samples containing 10%, 5% and 1% of mutated allele, respectively. Each calibration sample was prepared in duplicate. In addition, 8 known mosaic patients were also analyzed; they had been identified by Sanger sequencing of multiple tumors of each patient while blood DNAs sequencing did not confirm the variants found in tumor tissues in any of the patients.

Finally, we screened by deep sequencing a total of 34 consecutive DNA samples (4 tumor and 30 blood DNAs) referred to our laboratory for *NF2* mutational analysis. Fifteen patients had a NF2 clinical diagnosis based on the current diagnostic criteria [19–21]; the others had multiple schwannomas and/or meningiomas and/or ependimomas or 1 central nervous system tumor associated with juvenile cataract and/or cafè-au-lait spots (S1 Table). All the patients analyzed have been previously screened by MLPA analysis to exclude genomic rearrangements.

Data of the study (variants and phenotypes) were submitted at the Leiden Open Variation Database (LOVD; URL: http://lovd.nl/3.0/home).

Written informed consent was obtained from all subjects. The study was approved by the ethical committee of Careggi Hospital.

### Amplicon design and sequencing

Our amplicon design included *NF2* and *SMARCB1*(MIM 601607; NM_003073.3) genes; this choice was made for two main reasons: i) to increase the number of variants present in the calibration samples, and, ii) to use the same amplicon design to detect *SMARCB1* mutations in two other hereditary diseases, namely schwannomatosis (MIM162091) and Rhabdoid Tumor Predisposition Syndrome (MIM609322).

Selection of *NF2* and *SMARCB1* regions of interest (UTR+CDS) was performed by TruSeq Custom Amplicon (TSCA) protocol. Amplicons were designed by Illumina DesignStudio (http://www.illumina.com). A total of 44 amplicons of an average of 250 bp in length comprising about 5581 bp was obtained (100% on-target coverage). Library preparation was performed using the TruSeq Custom Amplicon Library Preparation Kit v1.5 (Illumina, San Diego, CA, USA). The genomic DNA input for amplicon library preparation was 250 ng for each sample according to manufacturer’s instructions. All sample libraries were equimolarly pooled and sequenced on the Illumina MiSeq Sequencer (Illumina, San Diego, CA, USA) with MiSeq Reagent Kit v2 (2X150 cycles) according to the manufacturer’s instructions.

### Data analysis and processing

Amplicon reads were aligned against the human reference genome hg19 with BWA MEM [22]. Alignments were soft-clipped based on forward and reverse primers length and position in each read by custom scripts. GATK version 2.5.2 [23] was used to recalibrate base qualities and realign aligned reads around indels. Regions with coverage less than or equal 100x in more than 50% of samples (low-coverage regions) were discarded for downstream analyses. A mosaic variant analysis was applied to detect low abundance variants: SNVs were identified using MuTect version 1.1.4 [16] with standard parameters and GATK IndelGenotyperV2 (with minFraction = 0.001 and minCnt = 3) was used to detect InDels. Genomic and functional annotation of detected variants was made by Annovar [24].

Coverage statistics was performed by DepthOfCoverage utility of GATK. BASH and R custom scripts were used to obtain the list of low coverage (≤100X) regions per sample.

### Statistical measures

A Receiver Operating Characteristic (ROC) curve analysis was used to estimate the variant allele fraction (VAF) to obtain the best detection accuracy for low level mosaicism identification. To this end, the known variants of calibration samples (Table 1) were exploited to perform this analysis. For each variant type analysis (SNVs and InDels) and for each calibration sample we estimated True Positive Rate (TPR*) and False Positive Rate (FPR*) at step *i* (*i*-th value of threshold parameter, that is VAF) as:

$$TPR^{*}{}_{i}=\frac{TP_{i}}{TP}=\;detection\;sensitivity$$

$$FPR^{*}{}_{i}=\frac{FP_{i}}{FP}=\;1-\;detection\;specificity$$

where *TP*_*i*_ and *FP*_*i*_ are respectively the number of true and false positive variants with *VAF* ≤ *VAF*_*i*_ while *TP* and *FP* are the total number of true and false positives (*TP* + *FP* is the total number of detected variants). Detection accuracy was estimated by measuring the Area Under the Curve (AUC) inferred from the ROC curves.

**Table 1:** Known variants present in calibration samples.

Gene^a^	Position(HG19)	Variant^b^	Samples	VariantType	Annotation
*NF2*	chr22:30000057	c.70_71insT	164	InDel	Frameshift insertion
*NF2*	chr22:30032783	c.158_165del	277	InDel	Frameshift deletion
*NF2*	chr22:30032794	c.169C>T	407	SNV	Stopgain
*NF2*	chr22:30050660	c.462delC	82	InDel	Frameshift insertion
*NF2*	chr22:30050662	c.464C>T	82	SNV	Nonsynonymous
*NF2*	chr22:30051658	c.592C>T	67	SNV	Stopgain
*SMARCB1*	chr22:24129129	c.-228G>T	277	SNV	rs11704810
*SMARCB1*	chr22:24129240	c.-117C>T	164, 67	SNV	rs11090285
*SMARCB1*	chr22:24167513	c.897G>A	277	SNV	rs2229354
*SMARCB1*	chr22:24176480	c.*113_*114insG	277	InDel	rs146383610

^*a*^: *NF2*: NM_181832.2; *SMARCB1*:NM_003073.3

^*b*^: The DNA variant numbering is based on cDNA sequences for both genes, with the A of the ATG translation-initiation codon numbered as +1.

### Standard PCR and HRMA Conditions

PCR reactions were performed in 20 ul, with 2uL 10X PCR buffer containing 1.5mM MgCl_2_ (Qiagen, Hilden Germany), 0.25 mM of each dNTP, 0.5 uM of forward and reverse primers, 0.5U HotStarTaq Plus (Qiagen, Hilden, Germany) and 100 ng of genomic DNA. SYTOs 9 (Invitrogen, Eugene, Oregon, USA) was used as the intercalating dye in HRMA. PCR condition consisted of an initial denaturation step at 95°C for 5 min, following by 35 cycles of denaturing at 95°C for 30 sec, 30 sec at annealing temperature and elongation at 72°C for 30 sec. The final extension was carried out at 72°C for 20 min for HRMA and 10 min for sequencing analysis.

HRM analysis was performed on the “Rotor Gene 6000 Instrument” (Corbett Research, Sydney, Australia). Amplified products were denaturated at 95°C for 1 min and then rapidly cooled to 40°C for 1 min in order to facilitate heteroduplex formation. Melt curve data for each PCR product were acquired in a wide temperature range (75°C to 95°C), at a ramping rate of 0.1°C/sec. Results were analyzed as fluorescence versus temperature graphs.

### COLD PCR

To design a COLD-PCR assay a new reduced denaturation temperature for the reactions was determined (Tc). The melting temperature (Tm) of a given amplicon was firstly identified via HRMA of conventional PCR using an intercalating dye: the Tc is usually 1°C below the experimentally derived Tm. Subsequently, a set of COLD-PCR reactions at graded temperatures below the Tm were performed, in order to identify the optimal Tc.

Fast COLD-PCR was used for the detection of Tm-decreasing variants and its cycling conditions were as follow: 95°C for 5 min; 20 cycles of 95°C for 30 sec, primer annealing temperature for 30 sec, 72°C for 30 sec; then 30 cycles of Tc for 3 sec, primer annealing temperature for 30 sec, 72°C for 30 sec; and final extension at 72°C for 10 min.

Full COLD-PCR protocol was applied for identifying Tm-increasing or-not modifying variants using the following conditions: denaturation at 95°C for 3 min, 25 cycles of 95°C for 15 sec, primers annealing temperature for 30 sec, 72°C for 1 min; then 30 cycles of 95°C for 15 sec, 70°C for 8 min, Tc for 3 sec, primers annealing temperature for 30 sec, 72°C for 1 min, and final extension at 72°C for 20 min. COLD-PCR assays were performed using the same final reagent concentration used in standard PCR. Primers sequences are available on request.

### Sanger Sequencing

PCR and COLD-PCR products were purified using HiYieldGel/PCR DNA Fragments Extraction Kit (RBCBioscience, New Taipei City, Taiwan). Sequencing analysis was performed on both strands using the BigDye Terminator v1.1 Cycle Sequencing Kit (Life Technologies Applied Biosystems, Austin, Texas, USA) and a model 310 automated sequencer (Life Technologies Applied Biosystems). Variant nomenclature follows the Human Genome Variation Society recommendations (http://www.hgvs.org). The DNA variant numbering is based on the *NF2* cDNA sequences (GenBank accession number NM_181832.2) with the A of the ATG translation-initiation codon numbered as +1.

### Multiplex Ligation Probe Amplification

Copy number changes (deletions or duplications) of *NF2* locus and flanking genes were analyzed by Multiplex Ligation-Dependent Probe Amplification (MLPA), using *NF2* and/or 22q11 MLPA test kits (MRC-Holland, P044_B1 and P324_A2). Electrophoresis data were analyzed by GeneMapper software (Life Technologies, Carlsbad, CA, USA).

## Results

### Coverage statistics

A mean number of 118842 reads were obtained for each sample. The mean median coverage per sample was 1121X ranging from 14X to 2045X (S2 Table and S1 Fig). Low-coverage regions represented a total of 467 bp (8.4% of target regions); however, the majority of them matched to intronic or UTR regions (346 bp, 75% of low-coverage regions), characterized by high GC content.

Low-coverage regions involving CDSs were a total of 121 bp: these included segments of exon 1 and exon 5 of *SMARCB1* and represented 4% of the total CDSs (2925 bp) of our design.

### Calibration samples analysis

Calibration samples were used to estimate the minimum Variant Allele Fraction (VAF) to reach the best analysis accuracy. S2 Fig reports the boxplots representing the correlation (R = 0.99) between expected and observed VAF. S3 Fig shows the electropherogram obtained by Sanger sequencing of the serial dilutions of the Calibration samples.

**Fig 1:**
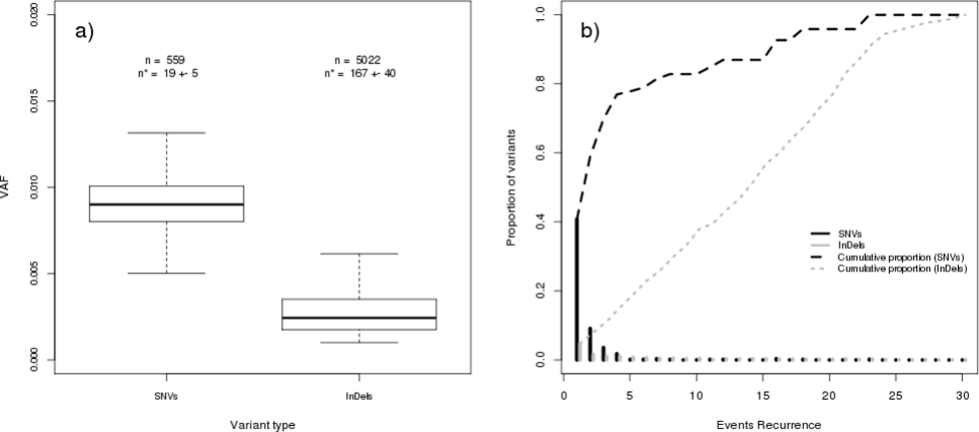
Features of false positives events detected in calibration samples. (a) Boxplot of VAFs of events detected by MuTect (SNVs) and IndelGenotyperV2 (InDels). n is the total number of detected events, n* is the mean number of events (per sample) with the corresponding standard deviation. (b) Histogram of events recurrence among calibration samples (n = 30) for SNVs (black) and InDels (grey). Corresponding cumulative percentages are reported in dashed black (SNVs) and grey (InDels) lines.

To understand the performance of our data analysis pipeline, we applied the mosaic variant analysis to calibration samples. Results for each calibration sample, summarized in S3 Table, show that False Positives (FPs) represent the great majority (~99%) of the detected variants. In addition, the great majority of them have very low allele frequency: only 10% of all the detected SNVs and InDels have allele frequency greater than 0.015 and 0.006, respectively (Fig 1A). Moreover, we observed that detected SNVs are mostly unique among samples: Fig 1B shows that ~40% of all SNVs are unique (Event recurrence = 1), while only 20% are present in more than 5 samples. On the other hand, detected InDels have a quasi-uniform recurrence: 50% of all of them are recurrent in more than 14 samples (Fig 1B).

**Fig 2:**
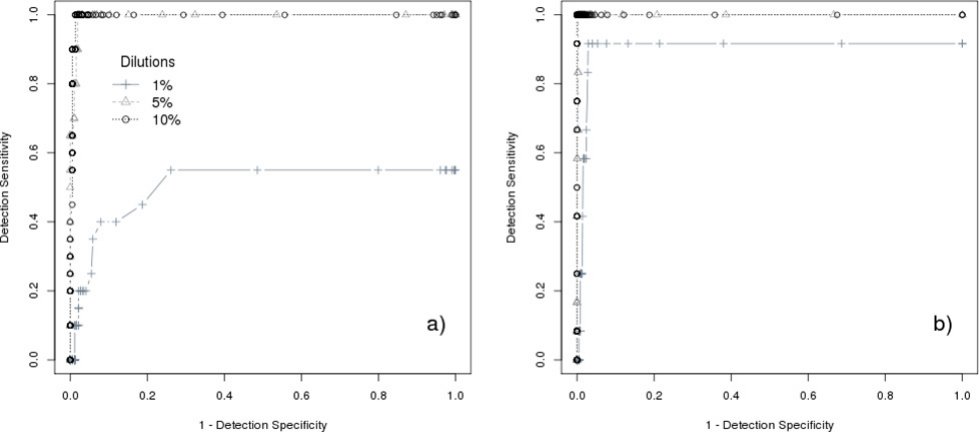
ROC curve analysis of variants found in calibration samples. ROC Curve analysis for SNVs (a) and InDels (b) events. Data are obtained by averaging results of calibration samples with the same dilution. Cross, triangle and circle points are relative to 1%, 5% and 10% dilution degree. Data do not contain recurrent events.

In order to improve the accuracy of our analysis, we used the estimated VAFs as threshold parameter to discriminate between false and true events. To this end, we used the variants detected by MuTect and IndelGenotyperV2, with their respective allele frequency, to perform a ROC curve analysis. To avoid introducing systematic errors in estimating statistical measures, recurrent events across all the calibration samples were discarded for downstream analysis. These events correspond to two InDels that have the same VAF in all the calibration samples, even for different dilutions (S4 Table). Fig 2 shows results of ROC curve analysis: for both SNVs (Fig 2A) and InDels (Fig 2B), we obtained a high accuracy for calibration samples with dilution equal to 5% and 10%, while detecting variants with VAF~1% remained challenging.

This is also shown in Table 2 that reports the statistical measures estimated from ROC curve analysis. At the estimated cut-off value, in samples with dilution 5% and 10% we are able to detect all the events (TPR > 0.99), drastically reducing the number of FPs events. On the contrary, in samples at 1% dilution, variants could be detected at the cost of specificity. In particular, we identified 15/22 variants (8/14 SNVs and 7/8 InDels), corresponding to a TPR = 0.55 for SNVs and TPR = 0.92 for InDels at the best VAF cut-off. In this case, we obtained about 5 false SNVs and 3 false InDels per sample.

**Table 2:** Results of ROC curve analysis on calibration samples.

Variant type	Dilution	Best VAF cut-off	FPR^a^	TPR^a^	Area under the ROC curve	Number of FPs^b^
SNV	0.01	0.01	0.26	0.55	0.5	5.5
	0.05	0.03	0.02	> 0.99	> 0.99	0.41
	0.1	0.06	0.01	> 0.99	> 0.99	0.28
InDel	0.01	0.01	0.02	0.92	0.91	2.8
	0.05	0.05	< 0.01	> 0.99	> 0.99	< 0.01
	0.1	0.08	< 0.01	> 0.99	> 0.99	< 0.01

^a^ TPR and FPR are calculated from ROC curves at the best VAF cut-off. This data do not contain recurrent events.

^b^ The number of FPs at each cut-off value was estimated by the product of FPR and the number of detected variants per sample

### Selection of candidate variants

ROC analysis results in calibration samples were exploited to select candidate mosaic variants in samples with unknown genotype. In particular, we used the estimated cut-off VAFs to filter detected variants. In order to control specificity, we required to obtain < 2 FP events (1 SNV and 1 InDel) per sample. This corresponds to select a cut-off VAF equal to 1.6% for both SNVs and InDels in all the ROC curves, irrespective of dilution (false positives are independent from dilutions). With this VAF value, we obtained TPR = 0.25 (for SNVs) and TPR = 0.58 (for InDels) in samples with dilution 1% and TPR > 0.99 for both SNVs and InDels in samples with dilution ≥ 5%.

Candidate variants were selected following the next step by step exclusion criteria:

1. variants included in dbSNP database or in 1000g project with frequency higher than 1%;
2. recurrent variants identified in more than 5 patients;
3. variants with VAF lower than 1.6%;
4. non synonymous variants predicted to be non-deleterious by at least two out of three open access prediction software questioned (PolyPhen 2, SIFT and Mutation Taster);
5. variants identified in samples carrying a pathogenetic variant confirmed by Sanger sequencing

The list of the candidate variants is reported in Table 3.

**Table 3:** Sample results obtained by filtering on the basis of VAF cut-off and functional criteria.

Sample	Type	Diagnosis	Median Coverage	Total InDels	Total SNVs	VAFfiltered InDels	VAFfiltered SNVs	Candidate variants^b^	VAF (%)	Sanger sequencing validation
11	B	mosaic NF2	813	159	18	1	2	-		-
65	B	mosaic NF2	442	81	12	3	2	c.193C>T^c^	2.03	-
106	B	mosaic NF2	1092	200	19	2	2	c.130_133del4insCACACGGGCGGC^c^	14.5	-
134	B	mosaic NF2	960	180	15	2	1	c.169C>T^c^	7.5	-
144	B	mosaic NF2	1289	214	30	0	1	c.586C>T^c^	1.95	-
241	B	mosaic NF2	628	109	21	1	4	c.592C>T^c^	3.7	-
295	B	mosaic NF2	891	174	19	3	2	c.1396C>T^c^	8.7	-
428	B	mosaic NF2	908	172	21	3	4	-	-	-
410	B	NF2	960	227	35	1	6	c.459C>A	2.4	YES^d^
451	B	NF2	1388	215	38	1	4	-	-	-
465	B	NF2	14	4	4	2	3	c.675+1G>T	39	YES
472	B	NF2	419	70	8	5	2	c.41_42del	19.2	YES
474	B	NF2	1243	179	28	1	1	-	-	-
481	B	NF2	728	146	17	1	2	c.52C>T	53.8	YES
484	B	NF2	1176	220	24	1	0	-	-	-
488	B	NF2	798	130	21	0	2	-	-	-
489	B	NF2	1363	193	20	0	0	-	-	-
491	B	NF2	1494	235	26	1	2	-	-	-
493	B	NF2	728	143	12	2	1	-	-	-
496	B	NF2	1092	194	17	0	3	-	-	-
500	B	NF2	2045	282	26	2	1	-	-	-
498	B	NF2	1494	248	42	2	3	c.448-2A>G	60	YES
492	B	NF2	1388	244	27	3	5	-	-	-
483	B	Uncertain	1033	220	21	4	1	c.916delC	50	YES
445	B	Uncertain	1764	264	25	2	3	-	-	-
453	B	Uncertain	1522	228	38	2	4	-	-	-
454	B	Uncertain	859	163	17	1	3	-	-	-
462	B	Uncertain	1033	203	23	3	3	-	-	-
463	B	Uncertain	1289	232	15	2	4	-	-	-
469_A	B	Uncertain	1522	259	35	4	1	-	-	-
469_B	B	Uncertain	1388	244	25	2	4	-	-	-
470	B	Uncertain	996	190	12	0	2	-	-	-
471	B	Uncertain	1414	218	18	1	3	-	-	-
478	B	Uncertain	1700	228	27	1	1	-	-	-
479	B	Uncertain	813	138	19	1	2	-	-	-
495	B	Uncertain	1764	249	18	1	3	-	-	-
497	B	Uncertain	1831	255	29	0	5	-	-	-
503	B	Uncertain	891	158	23	4	3	-	-	-
T754	T	NF2	1176	219	18	0	3	c.532C>T	29.2	YES
T819	T	NF2	828	139	11	2	2	c.448-2A>G	77.4	YES
T820	T	NF2	1092	184	20	4	2	c.448-2A>G	82.8	YES
T821	T	NF2	828	159	12	3	1	c.448-2A>G	78	YES

^a^: B: blood; T: tumor.

^b^:The DNA variant numbering is based on the *NF2* cDNA sequences (GenBank accession number NM_181832.2) with the A of the ATG translation-initiation codon numbered as +1.

^c^: Variants already characterized.

^d^: COLD-PCR protocol.

### Validation of verified mosaic patients

Verified mosaic patients were analyzed to confirm the presence and the VAF of each alteration. Our bioinformatic analysis correctly identified the variants in 6 out of 8 known mosaic samples. 7 blood DNAs showed VAF values under the Sanger sequencing detection limit, ranging from 8.7 to 1.95% (Table 3). In sample 106, the variant c.130_133delAAGGinsCACACGGGCGGC was identified with a VAF value of 14.5%; however, direct sequencing in blood DNA did not detect this alteration, maybe due to its complexity. Considering the high sensitivity of our method in detecting mosaic variants, the two remaining patients, whose mosaic diagnosis was made by the analysis of multiple tumors, likely, do not carry the alterations in blood cells.

### Sanger sequencing validation of *NF2* variants in samples with unknown *NF2* genotype

A total of 34 samples with unknown *NF2* genotype (30 blood DNAs and 4 tumor DNAs) were analyzed. Results obtained after filtering with the established VAF of 1.6% and the functional criteria, are reported in Table 3. Although its very low mean coverage, sample 465 was not excluded because we found a pathogenetic variant with high VAF (~ 39%), confirmed by direct sequencing.

**Fig 3:**
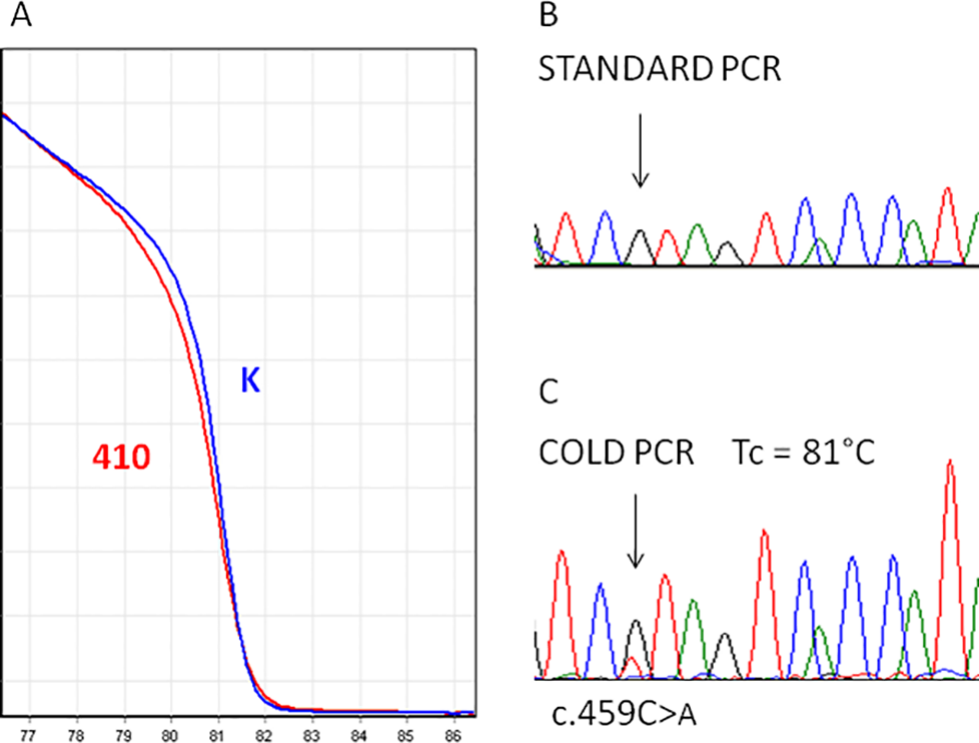
Validation of *NF2* variant in an unknown NF2 mosaic patient. The c.459C>T variant in exon 5 of the *NF2* gene was identified by deep sequencing. A) 410 DNA sample differed appreciably from wild-type melting curves at HRMA analysis; but the alteration was not detectable by Sanger sequencing after standard PCR (B). C) COLD-PCR allowed to enrich the variant allele as much as necessary to make the alteration clearly visible by Sanger sequencing.

*NF2* deleterious variants were identified in 6 out of 30 blood DNA samples (Table 3). Five DNA samples with a VAF ≥ 10%, were direct sequenced and variants were confirmed in all of them. Blood DNA sample from the last patient (410) showed a low abundance variant at deep sequencing analysis: the c.459C>A variant was detected with VAF of 2.4%. Direct sequencing of genomic DNA did not reveal any alteration; however, the sample showed a melting profile alteration in exon 5, at HRMA. Using fast COLD-PCR protocol, the mutated allele was selectively amplified as much as necessary to confirm the alteration using sequencing analysis (Fig 3).

We found *NF2* deleterious variants, confirmed by Sanger sequencing, in the four NF2-associated tumors (Table 3). Three tumors (T819, T820, T821) from patient 498, showed the same *NF2* variant, confirming it as a germline alteration. In the tumor DNAs, VAF (77.4%–82.8%) was substantially higher than the corresponding blood DNA. Indeed, at MLPA, the three tumors showed partial loss of 22q, involving the *NF2* gene.

The last tumor (T754) showed the c.532C>T variant, with a VAF of about 29%. This result is consistent with the maintenance of both copies of 22q, confirmed by MLPA analysis.

### Evaluation of variants with VAF lower than 1.6%

In order to assess whether the set VAF threshold would avoid losing detectable variants, we also evaluated variants with VAF < 1.6%. On the basis of the functional and clinical criteria, we selected two deleterious variants, identified in two different patients, 451 and 500.

In patient 451, the c.118A>T variant in exon 2 of *NF2* gene was pointed out with VAF of 1%. COLD PCR was optimized for detection of this alteration, using the full protocol, because the substitution A>T do not modify the melting temperature of the amplicon. However, the alteration was not confirmed by Sanger sequencing.

Patient 500 blood DNA showed the variant c.1396C>T, with VAF of 1% and direct sequencing of genomic DNA did not detect the alteration. COLD PCR conditions for c.1396C>T were previously optimized [15]. However, COLD PCR approach did not identify any alteration by Sanger sequencing.

## Discussion

Mosaicism is a frequent event (25–30%) in NF2 sporadic cases. The identification of mosaic variants in lymphocyte DNA of NF2 patients has important clinical implications and can optimize disease management. Indeed, the molecular diagnosis of these cases is fundamental to improve genetic counseling, mainly providing the chance to perform prenatal diagnosis.

Rapid advances in NGS technologies, together with the development of powerful computational tools, have open new scenarios in detecting mosaic variants. Amplicon sequencing can be used to increase low frequency fraction variant detection sensitivity by achieving a much higher read depth [25]. Nevertheless, the accurate detection of low-allelic variants is still challenging, particularly for the identification of somatic mosaicism, where matched control sample is absent.

To assess the capability in detecting low abundance variants in blood DNA, we screened by amplicon deep sequencing the UTRs and coding regions of *NF2* in a set of DNA samples referred to our laboratory with a medical request for *NF2* molecular analysis. MuTect and IndelGenotyperV2 were used to detect mosaic SNVs and InDels, respectively, and a ROC curve analysis was applied to estimate the minimum variant allele fraction (VAF) to obtain the best detection accuracy of low level mosaicism.

To figure out the accuracy of the method, we applied our data analysis pipeline for the detection of pathogenic variants in 30 calibration samples with known variants and VAF. We considered 10 different variants in 5 independent samples (Table 1), for a total of 42 SNVs and 24 InDels in all the calibration samples (different dilutions and replicates). Regarding sensitivity, we were able to identify 100% of the 46 known variants in samples with dilutions ≥ 5%. On the other hand, detecting events in samples with dilution 0.01 was challenging: we identified only 62% of the variants. Undetected variants either fall in amplicons with lower coverage or, even if they are supported by a high number of reads, their allele fraction is low (~0.01) (S5 Table). The latter case involves only SNVs events and is likely due to the downsampling strategy of MuTect. Regarding specificity, we had a large number of false detections (an average of 19 and 168 FPs for SNVs and InDels per sample, respectively) which constituted the great majority (> 99%) of all the detected variants.

Recently, Chen et al. [26] used a strategy based on calibration samples to identify low-level mosaic *RB1* variants in sporadic retinoblastoma cases. However, they did not report any evaluation on the detection accuracy of their method; in particular, data on the number of detected false positive events were missed. Our data show that estimating specificity in low level variants detection is fundamental to discern between true mosaicism and false events. For this reason, we studied the features of false positives identified in calibration samples. We observed (Fig 1A) that FPs had low VAF. Moreover, false InDels tend to be recurrent among different samples (Fig 1B). ROC curve analysis of calibration samples (Fig 2 and Table 2) allowed us to determine the VAF value that gave the best trade-off between sensitivity and specificity (best accuracy). We obtained very high accuracy in detecting variants with VAF **≥** 5%, while we found challenging to accurately detect variants with VAF~1%: for samples with dilution 1% at the estimated cut-off value of VAF we obtained 8 false detections that may be harder to validate, especially in diagnostic settings. For this reason, we selected a VAF cut-off to get < 2 FPs (1 for SNVs and 1 for InDels) per sample, obtaining VAF = 1.6 for both SNVs and InDels.

On the basis of the statistical results and functional criteria, we filtered the data from 34 samples with unknown *NF2* genotype (30 blood DNAs and 4 tumor DNAs) to identify causative alterations. A total of 6 variants were identified in blood DNAs with VAF ranging from 2.4% to 60%. DNA alterations with VAF higher than 10% were confirmed by direct sequencing. Moreover, we were able to detect causative *NF2* alterations in the analyzed tumors with VAF values consistent with the results obtained from MLPA analysis.

Our *NF2* variants detection rate in patients with a clear NF2 clinical diagnosis resulted of 33%.

*NF2* pathogenic alterations (point mutations and genomic rearrangements) can be found in about 45–55% of sporadic patients with bilateral vestibular schwannoma [2, 3]; considering that our group of patients had been previously analyzed by MLPA to rule out the presence of genomic rearrangements (10–15% of the *NF2* alterations), our detection rate is consistent with data from the literature.

In patient 410, deep sequencing identified the c.459C>A variant in *NF2* exon 5 with VAF of 2.4%. The low abundance alteration had not been detected by the analysis methods previously used: HRMA and Sanger sequencing of the coding and flanking regions of *NF2*. Patient 410 shows clinical features consistent with a diagnosis of mosaic NF2: bilateral vestibular schwannomas, one hypoglossal schwannoma and one falx cerebri meningioma, without involvement of peripheral nerves. The discovery, by deep sequencing, of the *NF2* pathogenic alteration in the patient allowed to set and optimize the COLD PCR conditions to enrich the minority allele, confirming the presence of the alteration by direct sequencing analysis. This result demonstrates that deep sequencing analysis associated to method for detecting low abundance variants, such as COLD- or Digital- PCR, may represent a suitable approach for increasing the diagnostic throughput in detecting mosaicism.

To evaluate if candidate variants with VAF < 1.6% could be detected by COLD PCR, we analyzed two potentially pathogenic variants, with VAF about of 1%, identified in two different patients. COLD PCR conditions were optimized for both variants, but none of them was verified by direct sequencing. This result confirmed that, in our experimental design, we could not discriminate between false or true positive variants for alterations with VAF < 1.6%. Likely, more sensitive methods, such as Digital PCR [27], could detect variants with lower VAF; however, the number of candidate alterations sharply increase when variants with VAF < 1.6% are included: about 8 variants (InDels and SNVs) per sample are found when the cut-off VAF is set at 1%. Optimizing conditions for COLD or Digital PCR is time consuming and the costs might become prohibitive if 8 alterations per sample have to be validated. Moreover, depending on their localization and type, some variants may still remain unconfirmed [13].

Noteworthy, the VAF cut-off used in this work is strictly related to our experimental design but the procedure we used is not. Actually, the method we propose (sequencing of serial dilutions of samples with known variants, followed by ROC curve analysis) is useful to define the VAF cut-offs when planning a study to detect low abundance variants, even though different NGS platforms and bioinformatic tools in investigating different genomic regions may lead to different performance in terms of detection accuracy.

In conclusion, the proposed method may be useful for optimizing every deep sequencing experimental plan. Indeed, regardless of the experimental design, the use of calibration samples followed by tailored statistical analysis enables to estimate the minimum allele fraction detectable with high accuracy, or, in other terms, to evaluate the background noise of deep sequencing analysis.

## Supporting Information

S1 Fig Boxplots reporting the median coverage in each targeted genomic region for all samples. (TIF)

S2 Fig Correlation between expected and observed allele fraction. Boxplots showing the correlation between expected and observed VAF calculated as the ratio between counts of reads with variant and wild-type alleles.
(TIFF)

S3 Fig Evaluation of sensitivity of Sanger sequencing in detecting mosaicism. We prepared dilutions of *NF2* mutated genomic samples (corresponding to 10%, 5% and 1% of mutated allele) with a wild type DNA. Sanger sequencing sensitivity was variable depending on the position and the type of alteration: the mutated allele was easy visible until the 10% dilution for the c.169C>T and c.592C>T substitutions and for the c.71_72insT insertion, while the ins/del c.161_168delTGGGGCT and c.463delCCinsT were barely detectable at 10% dilution. Sanger sequencing did not identify any of the variants at 1% dilution.
(TIF)

S1 Table Clinical data of patients. (DOCX)

S2 Table Details and technical sequencing data on sequenced samples. (DOC)

S3 Table Summary of variants identified in calibration samples. (DOC)

S4 Table Recurrent events that were excluded for downstream analysis. For each variant, mean, min (first values in parenthesis) and max (second values in parenthesis) values for each dilution are reported.
(DOC)

S5 Table Features of known variants in calibration samples with dilution 0.01. (DOC)
