# Supplementary figures and images for: A Systematic Assessment of Accuracy in Detecting Somatic Mosaic Variants by Deep Amplicon Sequencing: Application to NF2 Gene
Source: PLoS One. 2015 Jun 12;10(6):e0129099. doi: 10.1371/journal.pone.0129099 (PMC4466335; doi:10.1371/journal.pone.0129099)

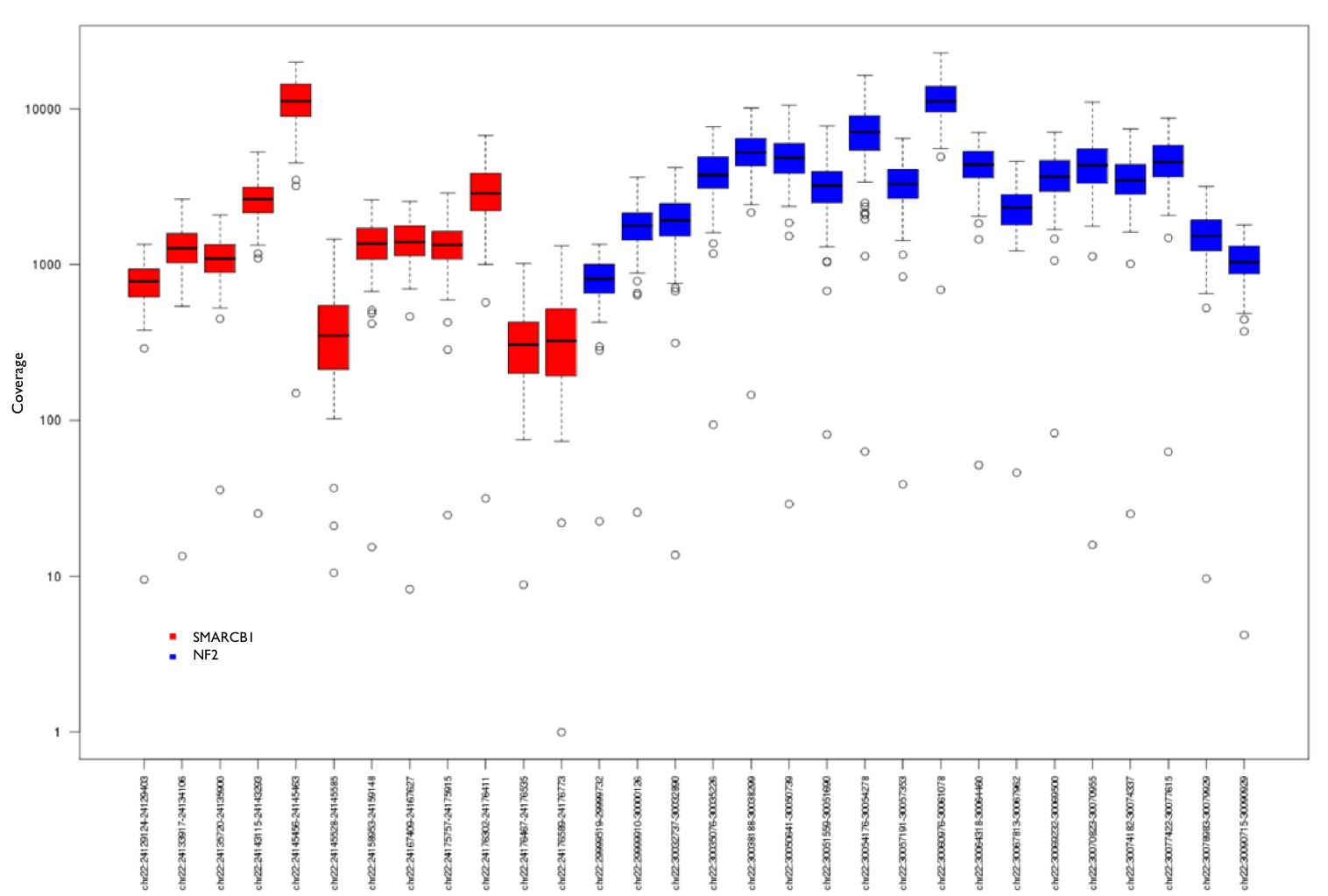

Supplement: S1 Fig — (TIF) [file pone.0129099.s001.tif]

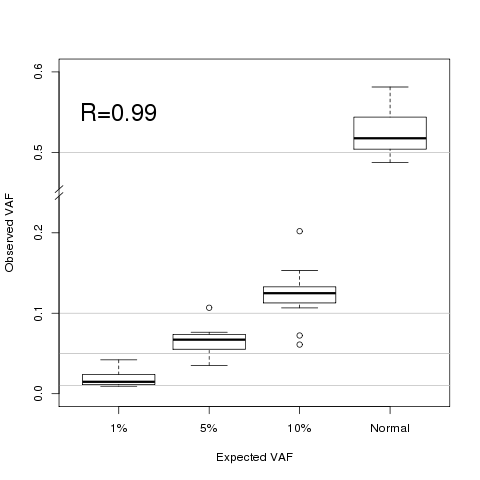

Supplement: S2 Fig — Boxplots showing the correlation between expected and observed VAF calculated as the ratio between counts of reads with variant and wild-type alleles. (TIFF) [file pone.0129099.s002.tiff]

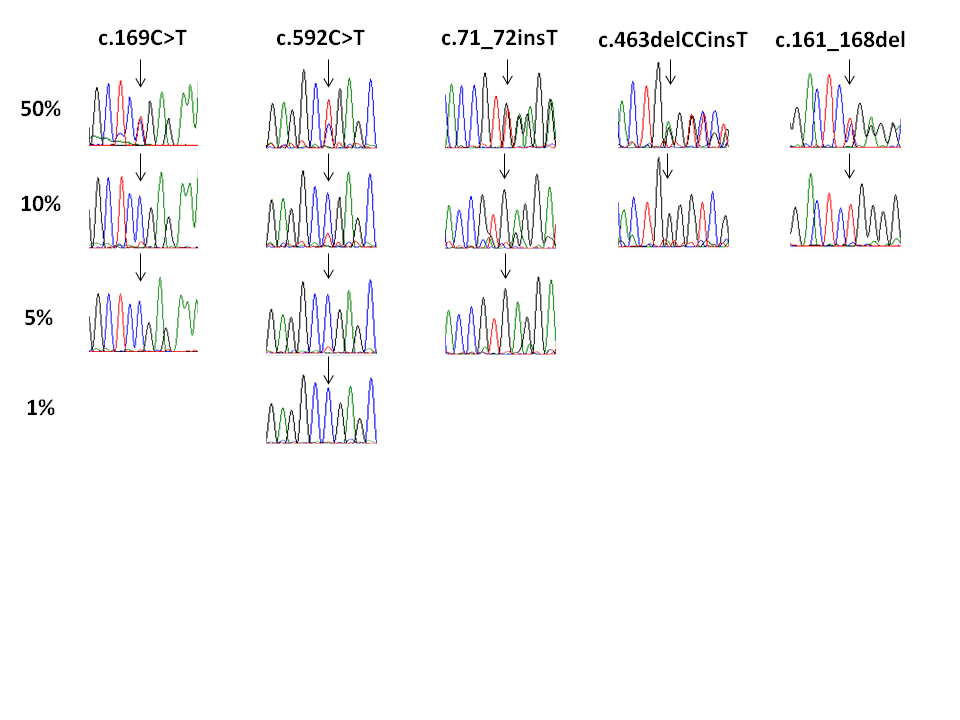

Supplement: S3 Fig — We prepared dilutions of NF2 mutated genomic samples (corresponding to 10%, 5% and 1% of mutated allele) with a wild type DNA. Sanger sequencing sensitivity was variable depending on the position and the type of alteration: the mutated allele was easy visible until the 10% dilution for the c.169C>T and c.592C>T substitutions and for the c.71_72insT insertion, while the ins/del c.161_168delTGGGGCT and c.463delCCinsT were barely detectable at 10% dilution. Sanger sequencing did not identify any of the variants at 1% dilution. (TIF) [file pone.0129099.s003.tif]
