# Supplementary material for: A Systematic Assessment of Accuracy in Detecting Somatic Mosaic Variants by Deep Amplicon Sequencing: Application to NF2 Gene
Source: PLoS One. 2015 Jun 12;10(6):e0129099. doi: 10.1371/journal.pone.0129099 (PMC4466335; doi:10.1371/journal.pone.0129099)
Supplement: S1 Table — (DOCX) [file pone.0129099.s004.docx]

| **Family** | **Case** | **Age** | **Sex^a^** | **Inheritance** | **Schwannomas** | | **meningiomas** | **Ependimomas** | **Cataracts** | **CLS** | **Clinical Criteria** | | | **NF2 Mutation** |
| --- | --- | --- | --- | --- | --- | --- | --- | --- | --- | --- | --- | --- | --- | --- |
|  |  |  |  |  | **Vestibular** | **other nerves** |  |  |  |  | **NIH** | **Manchester** | **NNFF** |  |
| 472 | 472IV | 34 | M | Sporadic | BVS | 0 | 2 | 0 |  | 0 | NF2 | NF2 | Definite NF2 | Yes |
| 481 | 481DBFS | 34 | M | Sporadic | BVS | 2 | 2 | 0 | Monolateral | 0 | NF2 | NF2 | Definite NF2 | Yes |
| 491 | 491BF | 48 | M | Sporadic | BVS | 1 | 0 | 0 |  | 0 | NF2 | NF2 | Definite NF2 |  |
| 493 | 493MM | 64 | F | Sporadic | BVS | 0 | 0 | 0 |  | 0 | NF2 | NF2 | Definite NF2 |  |
| 489 | 489TMC | 31 | F | Sporadic | BVS | 0 | Multiple | 0 |  | 0 | NF2 | NF2 | Definite NF2 |  |
| 488 | 488QC | 23 | F | Sporadic | BVS | 1 | 0 | 0 |  | 0 | NF2 | NF2 | Definite NF2 |  |
| 484 | 484CP | 43 | M | Sporadic | BVS | 0 | 0 | 0 |  | 0 | NF2 | NF2 | Definite NF2 |  |
| 474 | 474GF | 42 | F | Sporadic | BVS | 0 | 0 | 0 |  |  | NF2 | NF2 | Definite NF2 |  |
| 410 | 410EM | 33 | M | Sporadic | BVS | 1 | 1 | 0 |  | 0 | NF2 | NF2 | Definite NF2 | Yes |
| 498 | 498AAA | 38 | M | Sporadic | BVS | 0 | Multiple | 1 |  |  | NF2 | NF2 | Definite NF2 | Yes |
| 451 | 451CJ | 30 | F | Sporadic | UVS | 2 | 1 |  |  | 2 | NF2 | NF2 | Probable NF2 |  |
| 465 | 465CB | 8 | F | Sporadic | UVS | 1 | 1 | 0 |  | Yes | NF2 | NF2 | Probable NF2 | Yes |
| 496 | 496MA | 33 | M | Sporadic | UVS | 0 | Multiple | 1 |  | 0 | NF2 | NF2 |  |  |
| 500 | 500BR | 54 | F | Sporadic | UVS | 0 | 2 | 0 |  | 0 |  | NF2 |  |  |
| 492 | 492CL | 48 | M | Sporadic | NO | 2 | 2 | 0 |  | 0 |  | NF2 | Probable NF2 |  |
| 483 | 483CD | 14 | M | Sporadic | NO | 4 | 1 | 0 | Bilateral | 0 |  |  |  | Yes |
| 478 | 478DFG | 27 | F | Sporadic | UVS | 0 | 0 | 1 |  | 0 |  |  |  |  |
| 503 | 503ZA | 38 | F | Sporadic | UVS | 0 | 0 | 1 |  | 0 |  |  |  |  |
| 454 | 454GS | 46 | M | Sporadic | NO | 0 | Multiple | 1 |  | 0 |  |  |  |  |
| 469 | 469BV | 51 | M | Sporadic | UVS | 0 | 0 | 0 |  | Yes |  |  |  |  |
| 469 | 469BS | 16 | F | Familial | NO | 0 | 0 | 0 |  | Yes |  |  |  |  |
| 497 | 497PA | 56 | F | Sporadic | UVS | 0 | 0 | 0 |  | Yes |  |  |  |  |
| 462 | 462PM | 38 | M | Sporadic | UVS | 0 | 1 (malignant) | 0 |  | 0 |  |  |  |  |
| 479 | 479TA | 40 | F | Sporadic | UVS | 0 | 0 | 0 | Bilateral | 0 |  |  |  |  |
| 453 | 453CL | 32 | F | Sporadic | NO | 1 | 1 |  |  | 0 |  |  |  |  |
| 470 | 470CG | 57 | M | Sporadic | NO | 0 | 0 | 2 |  | 2 |  |  |  |  |
| 471 | 471PA | 42 | F | Sporadic | NO | 0 | 1 | 0 |  | 2 |  |  |  |  |
| 495 | 495CA | 13 | F | Sporadic | NO | 1 | 0 | 0 |  | Yes |  |  |  |  |
| 445 | 445TR | 16 | F | Sporadic | NO | 0 | 0 | 0 | Bilateral | >6 |  |  |  |  |
| 463 | 463BA | 46 | F | Sporadic | NO | 1 | 1 | 0 |  | 0 |  |  |  |  |

**S1 Table:** Clinical data of patients.

^a^ F= female; M= male
