# Supplementary material for: A Systematic Assessment of Accuracy in Detecting Somatic Mosaic Variants by Deep Amplicon Sequencing: Application to NF2 Gene
Source: PLoS One. 2015 Jun 12;10(6):e0129099. doi: 10.1371/journal.pone.0129099 (PMC4466335; doi:10.1371/journal.pone.0129099)
Supplement: S2 Table — (DOC) [file pone.0129099.s005.doc]

**S2 Table**: Details and technical sequencing data on sequenced samples.

| **Sample** | **Sample Type** | **Mean Coverage** | **Median coverage** | **Total reads** | **% bases above 0** | **% bases above 20** | **% bases with Phred score > 30** | **% reads with mapping quality > 20** |
| --- | --- | --- | --- | --- | --- | --- | --- | --- |
| 164 | Calibrator | 1565 | 1220 | 129030 | 100 | 99 | 98.9 | 99.6 |
| 164-10X-1 | Calibrator | 844 | 689 | 72466 | 100 | 94.3 | 99.1 | 99.6 |
| 164-10X-2 | Calibrator | 1221 | 925 | 102046 | 100 | 96.6 | 98.7 | 99.6 |
| 164-1X-1 | Calibrator | 1517 | 1092 | 127420 | 100 | 100 | 98.6 | 99.4 |
| 164-1X-2 | Calibrator | 1705 | 1313 | 143134 | 100 | 100 | 99.3 | 99.5 |
| 164-5X-1 | Calibrator | 1379 | 1052 | 116740 | 100 | 97.7 | 98.5 | 99.5 |
| 164-5X-2 | Calibrator | 1655 | 1388 | 138392 | 100 | 100 | 99.1 | 99.5 |
| 277 | Calibrator | 1296 | 1033 | 107958 | 100 | 98.9 | 98.9 | 99.4 |
| 277-10X-1 | Calibrator | 1609 | 1220 | 133698 | 100 | 100 | 98.4 | 99.6 |
| 277-10X-2 | Calibrator | 1585 | 1243 | 132614 | 100 | 100 | 99.0 | 99.5 |
| 277-1X-1 | Calibrator | 820 | 573 | 68258 | 100 | 94.3 | 99.0 | 99.5 |
| 277-1X-2 | Calibrator | 962 | 689 | 81018 | 100 | 96.6 | 98.9 | 99.4 |
| 277-5X-1 | Calibrator | 1623 | 1220 | 135358 | 100 | 100 | 99.1 | 99.5 |
| 277-5X-2 | Calibrator | 622 | 442 | 51764 | 100 | 91.1 | 99.0 | 99.4 |
| 407 | Calibrator | 433 | 323 | 36908 | 100 | 86.8 | 98.9 | 99.5 |
| 407-10X-1 | Calibrator | 1010 | 701 | 83182 | 100 | 95.4 | 98.6 | 99.6 |
| 407-10X-2 | Calibrator | 1329 | 978 | 110884 | 100 | 96.6 | 99.0 | 99.4 |
| 407-1X-1 | Calibrator | 1356 | 960 | 111600 | 100 | 98.7 | 98.7 | 99.6 |
| 407-1X-2 | Calibrator | 1366 | 942 | 112520 | 100 | 96.7 | 98.9 | 99.5 |
| 407-5X-1 | Calibrator | 1261 | 978 | 103822 | 100 | 96.6 | 99.1 | 99.5 |
| 407-5X-2 | Calibrator | 1580 | 1112 | 130532 | 100 | 98.8 | 99.2 | 99.5 |
| 67 | Calibrator | 1604 | 1176 | 133670 | 100 | 100 | 98.8 | 99.6 |
| 67-10X-1 | Calibrator | 1291 | 908 | 107764 | 100 | 100 | 99.1 | 99.4 |
| 67-10X-2 | Calibrator | 1609 | 1133 | 134080 | 100 | 100 | 99.5 | 99.5 |
| 67-1X-1 | Calibrator | 1255 | 891 | 104558 | 100 | 100 | 98.9 | 99.4 |
| 67-1X-2 | Calibrator | 1255 | 942 | 103970 | 100 | 100 | 99.1 | 99.5 |
| 67-5X-1 | Calibrator | 1203 | 891 | 100666 | 100 | 96.6 | 99.4 | 99.6 |
| 67-5X-2 | Calibrator | 1220 | 843 | 101114 | 100 | 97.9 | 98.9 | 99.4 |
| 82 | Calibrator | 944 | 676 | 79352 | 100 | 96.6 | 99.0 | 99.5 |
| 82-10X-1 | Calibrator | 1594 | 1112 | 133178 | 100 | 100 | 98.7 | 99.4 |
| 82-10X-2 | Calibrator | 839 | 628 | 70286 | 100 | 95.4 | 99.1 | 99.5 |
| 82-1X-1 | Calibrator | 1253 | 942 | 104238 | 100 | 100 | 98.8 | 99.5 |
| 82-1X-2 | Calibrator | 1236 | 908 | 102200 | 100 | 98.8 | 99.0 | 99.4 |
| 82-5X-1 | Calibrator | 1231 | 891 | 103304 | 100 | 96.6 | 99.3 | 99.5 |
| 82-5X-2 | Calibrator | 1081 | 755 | 90102 | 100 | 98.8 | 98.8 | 99.6 |
| 106 | Known mosaic | 1492 | 1092 | 120998 | 100 | 100 | 99.0 | 99.5 |
| 11 | Known mosaic | 1176 | 813 | 97052 | 100 | 97.7 | 98.9 | 99.4 |
| 134 | Known mosaic | 1287 | 960 | 106852 | 100 | 100 | 98.7 | 99.5 |
| 144 | Known mosaic | 1630 | 1289 | 133698 | 100 | 100 | 98.7 | 99.4 |
| 241 | Known mosaic | 812 | 628 | 68302 | 100 | 89.2 | 98.9 | 99.5 |
| 295 | Known mosaic | 1226 | 891 | 100136 | 100 | 96.2 | 98.9 | 99.6 |
| 428 | Known mosaic | 1188 | 908 | 98296 | 100 | 99 | 98.6 | 99.6 |
| 65 | Known mosaic | 632 | 442 | 52010 | 100 | 93.2 | 98.7 | 99.6 |
| 410 | Unknown | 1396 | 960 | 113974 | 100 | 99 | 99.1 | 99.5 |
| 445 | Unknown | 2285 | 1764 | 191424 | 100 | 100 | 98.8 | 99.5 |
| 451 | Unknown | 1781 | 1388 | 143806 | 100 | 100 | 99.2 | 99.4 |
| 453 | Unknown | 2038 | 1522 | 166876 | 100 | 100 | 98.9 | 99.6 |
| 454 | Unknown | 1040 | 859 | 84894 | 100 | 100 | 98.6 | 99.4 |
| 462 | Unknown | 1390 | 1033 | 113760 | 100 | 100 | 98.4 | 99.5 |
| 463 | Unknown | 1722 | 1289 | 140934 | 100 | 99 | 98.6 | 99.6 |
| 465 | Unknown | 35 | 14 | 3262 | 93.7 | 2.36 | 98.8 | 99.0 |
| 469_A | Unknown | 1982 | 1522 | 164800 | 100 | 100 | 99.0 | 99.4 |
| 469_B | Unknown | 1774 | 1388 | 143154 | 100 | 100 | 99.0 | 99.5 |
| 470 | Unknown | 1334 | 996 | 108378 | 100 | 100 | 98.7 | 99.5 |
| 471 | Unknown | 1997 | 1414 | 164254 | 100 | 100 | 98.6 | 99.6 |
| 472 | Unknown | 559 | 419 | 46122 | 100 | 93.4 | 98.9 | 99.6 |
| 474 | Unknown | 1499 | 1243 | 121792 | 100 | 97.9 | 98.9 | 99.4 |
| 478 | Unknown | 2113 | 1700 | 172186 | 100 | 100 | 99.1 | 99.5 |
| 479 | Unknown | 993 | 813 | 82852 | 100 | 97.6 | 98.6 | 99.4 |
| 481 | Unknown | 917 | 728 | 76368 | 100 | 96.2 | 98.4 | 99.6 |
| 483 | Unknown | 1343 | 1033 | 111002 | 100 | 97.6 | 98.5 | 99.6 |
| 484 | Unknown | 1498 | 1176 | 121790 | 100 | 100 | 99.4 | 99.5 |
| 488 | Unknown | 1020 | 798 | 84768 | 100 | 96.8 | 99.0 | 99.5 |
| 489 | Unknown | 1687 | 1363 | 139680 | 100 | 100 | 98.7 | 99.4 |
| 491 | Unknown | 1965 | 1494 | 161592 | 100 | 100 | 98.5 | 99.4 |
| 492 | Unknown | 1982 | 1388 | 165372 | 100 | 100 | 98.8 | 99.5 |
| 493 | Unknown | 970 | 728 | 80436 | 100 | 99 | 99.3 | 99.5 |
| 495 | Unknown | 2385 | 1764 | 197232 | 100 | 97.6 | 98.9 | 99.6 |
| 496 | Unknown | 1496 | 1092 | 122848 | 100 | 99 | 99.4 | 99.6 |
| 497 | Unknown | 2372 | 1831 | 195346 | 100 | 100 | 99.0 | 99.4 |
| 498 | Unknown | 2099 | 1494 | 173656 | 100 | 100 | 98.5 | 99.6 |
| 500 | Unknown | 2659 | 2045 | 219938 | 100 | 100 | 99.2 | 99.6 |
| 503 | Unknown | 1187 | 891 | 98968 | 100 | 96 | 98.9 | 99.6 |
| T754^a^ | Unknown | 1480 | 1176 | 123460 | 100 | 100 | 98.9 | 99.5 |
| T819 ^a^ | Unknown | 1124 | 828 | 94918 | 100 | 97.1 | 99.3 | 99.4 |
| T820 ^a^ | Unknown | 1406 | 1092 | 117992 | 100 | 100 | 98.8 | 99.5 |
| T821 ^a^ | Unknown | 1026 | 828 | 86796 | 100 | 99 | 98.5 | 99.5 |

^a^DNA extracted from tumor tissues.
