# Supplementary material for: A Systematic Assessment of Accuracy in Detecting Somatic Mosaic Variants by Deep Amplicon Sequencing: Application to NF2 Gene
Source: PLoS One. 2015 Jun 12;10(6):e0129099. doi: 10.1371/journal.pone.0129099 (PMC4466335; doi:10.1371/journal.pone.0129099)
Supplement: S3 Table — (DOC) [file pone.0129099.s006.doc]

**S3 Table:** Summary of variants identified in calibration samples.

| **Sample** | **Dilution** | **Total SNVs** | **TP SNVs** | **FP SNVs** | **Total InDels** | **TP InDels** | **FP InDels** |
| --- | --- | --- | --- | --- | --- | --- | --- |
| 407-1X-1 | 1X | 18 | 1 | 17 | 167 | 0 | 167 |
| 407-1X-2 | 1X | 21 | 0 | 21 | 168 | 0 | 168 |
| 407-5X-1 | 5X | 17 | 1 | 16 | 158 | 0 | 158 |
| 407-5X-2 | 5X | 20 | 1 | 19 | 189 | 0 | 189 |
| 407-10X-1 | 10X | 11 | 1 | 10 | 113 | 0 | 113 |
| 407-10X-1 | 10X | 11 | 1 | 10 | 161 | 0 | 161 |
| 277-1X-1 | 1X | 18 | 1 | 17 | 120 | 2 | 118 |
| 277-1X-2 | 1X | 20 | 2 | 18 | 140 | 1 | 139 |
| 277-5X-1 | 5X | 33 | 2 | 31 | 218 | 2 | 216 |
| 277-5X-2 | 5X | 9 | 2 | 7 | 88 | 2 | 86 |
| 277-10X-1 | 10X | 27 | 2 | 25 | 245 | 2 | 243 |
| 277-10X-2 | 10X | 24 | 2 | 22 | 222 | 2 | 220 |
| 164-1X-1 | 1X | 21 | 1 | 20 | 196 | 1 | 195 |
| 164-1X-2 | 1X | 30 | 0 | 30 | 213 | 1 | 212 |
| 164-5X-1 | 5X | 15 | 1 | 14 | 156 | 1 | 155 |
| 164-5X-2 | 5X | 21 | 1 | 20 | 193 | 1 | 192 |
| 164-10X-1 | 10X | 21 | 1 | 20 | 104 | 1 | 103 |
| 164-10X-2 | 10X | 21 | 1 | 20 | 165 | 1 | 164 |
| 67-1X-1 | 1X | 17 | 1 | 16 | 166 | 0 | 166 |
| 67-1X-2 | 1X | 14 | 1 | 13 | 176 | 0 | 176 |
| 67-5X-1 | 5X | 21 | 2 | 19 | 164 | 0 | 164 |
| 67-5X-2 | 5X | 26 | 2 | 24 | 165 | 0 | 165 |
| 67-10X-1 | 10X | 24 | 2 | 22 | 188 | 0 | 188 |
| 67-10X-2 | 10X | 20 | 2 | 18 | 220 | 0 | 220 |
| 82-1X-1 | 1X | 16 | 0 | 16 | 171 | 1 | 170 |
| 82-1X-2 | 1X | 26 | 1 | 25 | 162 | 1 | 161 |
| 82-5X-1 | 5X | 20 | 1 | 19 | 153 | 1 | 152 |
| 82-5X-2 | 5X | 16 | 1 | 15 | 133 | 1 | 132 |
| 82-10X-1 | 10X | 23 | 1 | 22 | 226 | 1 | 225 |
| 82-10X-2 | 10X | 14 | 1 | 13 | 118 | 1 | 117 |
