# Supplementary material for: A Systematic Assessment of Accuracy in Detecting Somatic Mosaic Variants by Deep Amplicon Sequencing: Application to NF2 Gene
Source: PLoS One. 2015 Jun 12;10(6):e0129099. doi: 10.1371/journal.pone.0129099 (PMC4466335; doi:10.1371/journal.pone.0129099)
Supplement: S4 Table — For each variant, mean, min (first values in parenthesis) and max (second values in parenthesis) values for each dilution are reported. (DOC) [file pone.0129099.s007.doc]

**S4 Table:** Recurrent events that were excluded for downstream analysis. For each variant, mean min (first values in parenthesis) and max (second values in parenthesis) values, for each dilution, are reported.

| Variant | VAF (Normal)  mean (min, max) | VAF (10X)  mean (min, max) | VAF (5X)  mean (min, max) | VAF (1X)  mean (min, max) |
| --- | --- | --- | --- | --- |
| NM_181832:c.599+36delG | 0.028  (0.024, 0.032) | 0.026  (0.018, 0.036) | 0.025  (0.019, 0.035) | 0.023  (0.012, 0.035) |
| NM_003073:c.-165insGCG | 0.040  (0.032, 0.044) | 0.053  (0.045, 0.074) | 0.055  (0.042, 0.070) | 0.059  (0.036, 0.064) |
