# Supplementary material for: A Systematic Assessment of Accuracy in Detecting Somatic Mosaic Variants by Deep Amplicon Sequencing: Application to NF2 Gene
Source: PLoS One. 2015 Jun 12;10(6):e0129099. doi: 10.1371/journal.pone.0129099 (PMC4466335; doi:10.1371/journal.pone.0129099)
Supplement: S5 Table — (DOC) [file pone.0129099.s008.doc]

**S5 Table**: Features of known variants in calibration samples with dilution 0.01.

| **Gene** | **Variant^a^** | **Samples** | **Variant Type** | **Detected** | **Total Coverage** | **Variant Coverage** | **VAF** |
| --- | --- | --- | --- | --- | --- | --- | --- |
| *NF2* | c.70_71insT | 164-1X-1 | indel | YES | 277 | 7 | 0.025 |
| *NF2* | c.70_71insT | 164-1X-2 | indel | YES | 287 | 5 | 0.017 |
| *NF2* | c.158_165del | 277-1X-1 | indel | YES | 538 | 6 | 0.011 |
| *NF2* | c.158_165del | 277-1X-2 | indel | YES | 673 | 8 | 0.012 |
| *NF2* | c.169C>T | 407-1X-1 | SNV | YES | 730 | 8 | 0.011 |
| *NF2* | c.169C>T | 407-1X-2 | SNV | NO | 842 | 5 | 0.006 |
| *NF2* | c.462delC | 82-1X-1 | indel | YES | 1371 | 15 | 0.011 |
| *NF2* | c.462delC | 82-1X-2 | indel | YES | 1318 | 22 | 0.017 |
| *NF2* | c.464C>T | 82-1X-1 | SNV | NO | 1371 | 15 | 0.011 |
| *NF2* | c.464C>T | 82-1X-2 | SNV | YES | 1318 | 16 | 0.012 |
| *NF2* | c.592C>T | 67-1X-1 | SNV | YES | 1352 | 23 | 0.017 |
| *NF2* | c.592C>T | 67-1X-2 | SNV | YES | 1495 | 47 | 0.031 |
| *SMARCB1* | c.-228G>T | 277-1X-1 | SNV | NO | 524 | 6 | 0.011 |
| *SMARCB1* | c.-228G>T | 277-1X-2 | SNV | YES | 667 | 16 | 0.024 |
| *SMARCB1* | c.-117C>T | 164-1X-1 | SNV | YES | 216 | 10 | 0.046 |
| *SMARCB1* | c.-117C>T | 164-1X-2 | SNV | NO | 250 | 5 | 0.020 |
| *SMARCB1* | c.-117C>T | 67-1X-1 | SNV | NO | 178 | 0 | 0.000 |
| *SMARCB1* | c.-117C>T | 67-1X-1 | SNV | NO | 187 | 3 | 0.016 |
| *SMARCB1* | c.897G>A | 277-1X-1 | SNV | YES | 1325 | 21 | 0.016 |
| *SMARCB1* | c.897G>A | 277-1X-2 | SNV | YES | 1449 | 16 | 0.011 |
| *SMARCB1* | c.*113insG | 277-1X-1 | indel | YES | 90 | 4 | 0.044 |
| *SMARCB1* | c.*113insG | 277-1X-2 | indel | NO | 72 | 2 | 0.028 |

^a^ The DNA variant numbering is based on the cDNA sequences (GenBank accession number *NF2*: NM_181832.2 and *SMARCB1*: NM_ 003073) with the A of the ATG translation-initiation codon numbered as+1.
